# Laypersons' Assessment of Smile Esthetics in Young Individuals in Ha'il, Saudi Arabia

**DOI:** 10.7759/cureus.60726

**Published:** 2024-05-20

**Authors:** Mohammad D Aljanakh, Wejdan N Alshammari, Khalid A Aljameel, Alanoud S Alshammari, Ibrahim R Altheban, Najd S Alzaid, Sami Almohefer

**Affiliations:** 1 Department of Restorative Dentistry, College of Dentistry, University of Ha'il, Ha’il, SAU; 2 Department of Restorative Dentistry, College of Dentistry, University of Ha'il, Ha'il, SAU

**Keywords:** laypersons' assessment, dental esthetics, attractiveness, smile analysis, smile esthetics

## Abstract

Introduction: When planning esthetic dental treatments, understanding smile preferences is important for dental professionals. This study aimed to evaluate the impact of selected smile characteristics on the attractiveness of young Saudis as assessed by Saudi laypersons and explore gender-preferred changes in smile attractiveness.

Methodology: This observational study assessed the dynamic smile attractiveness of 168 Saudi individuals (84 males and 84 females), selected through non-probability convenience sampling. Dynamic smiles were elicited by viewing comedic content and captured with a camera standardized for consistent positioning. Videos were edited and adjusted to images, and the frames with the most pronounced smiles were chosen. The intra-rater reliability was assessed using intra-class correlation coefficients (ICCs) and Cohen’s kappa tests (κ). The highest and lowest 25th percentiles were categorized as attractive and unattractive smiles, respectively, on the visual analog scale (VAS) by laypersons. Six smile characteristics - anterior smile line, smile arc, upper lip curvature, posterior teeth displayed, smile index, and smile symmetry - were quantitatively evaluated from these images for each participant and classified into attractive and unattractive groups based on laypersons' VAS evaluations. Continuous variables were tested with the Mann-Whitney U test, and for the categorical variables, the Chi-square test was applied. The significance was set at 5%.

Results: The four randomly selected out of the 22 raters had good VAS reliability; ICCs varied from 0.661 to 0.94, with an average of 0.737, and Cohen's kappa tests for smile characteristics showed values from 0.617 to 0.89. Good agreement was also found with the smile index, with ICCs of 0.775, and dynamic smile symmetry, with ICCs of 0.872. Laypersons rated female smiles as more attractive compared to male smiles (*P* = 0.004). Low or average anterior smile lines (*P* = 0.001 for males; *P* = 0.03 for females), parallel smile arcs (*P* = 0.001 for males; *P* = 0.02 for females), and higher smile indexes (*P* = 0.001 for males; *P* = 0.004 for females) were significantly attractive, showing no significant gender differences.

Conclusions: Laypersons reliably rated the young Saudis' dynamic smiles as attractive. Of the rated smile characteristics, those with a low or average anterior smile line, parallel smile arcs, and a larger smile index were deemed more attractive. This study's findings show no significant gender differences in the impact of the studied smile characteristics on attractiveness. This study's findings can help dental professionals customize treatment plans that meet patients' expectations.

## Introduction

Smiling is a fundamental aspect of social interactions and personal expression as it is the most apparent expression of emotion and an individual’s personality [1]. It occurs as an outcome of the complicated interrelationships with the teeth, supporting dental structures, and facial soft tissue [2]. Attractive smiles were associated with positive impressions and regarded as more friendly, intelligent, and appealing, affecting social acceptance, self-esteem, and life satisfaction [3,4]. Therefore, comprehending the factors that shape the perception of smile attractiveness is essential in aesthetic dentistry, as it directs the diagnosis and treatment of esthetic smiles.

Even though laypeople do not have the same sensitivity to assessing the dentoalveolar complex as dental professionals, they can recognize most factors that reduce smile attractiveness [5-9]. However, the preferences of laypersons are important as they are the main evaluators of dental esthetics. Therefore, recognizing these preferences can help clinicians in esthetic treatment planning.

Many studies have analyzed different aspects of smiles that influence how their attractiveness is perceived, including the shape and size of teeth, their proportion, position of the incisors, exposure, and shape of the maxillary gingival morphology, midline deviation, buccal corridor, the arc of the smile, smile index, and smile symmetry [10-12]. However, the impact of smile characteristics on smile attractiveness was less studied [13].

It is fundamental when evaluating the smile attractiveness to consider individual and cultural characteristics that can influence the attractiveness perception [14]. However, published works that explore the Saudi population do not provide a coherent view of the factors influencing the attractiveness of a smile [15]. The published studies in Saudi Arabia to explore the impact of smile characteristics on smile attractiveness were limited. These studies were conducted through a different methodology, none of them used dynamic smile analysis [16-19]. Therefore, this study was conducted to fill this gap.

This study aimed to evaluate the impact of selected smile characteristics on the attractiveness of young Saudis as rated by Saudi laypersons and to investigate any differences based on gender. Understanding these preferences can assist dental professionals in achieving the aesthetic expectations of this population and allow these professionals to customize esthetic dental treatment in this cultural context.

## Materials and methods

Study design and setting

An observational descriptive study was conducted at the University of Ha'il, Ha'il City, northern Saudi Arabia, from February to September 2023. 

Recruitment criteria and sampling

A convenient, non-probability sample of University of Ha'il students was selected. Based on previous similar studies, the minimal sample size for each attractive and unpleasant grin category was *N* = 18, with 80% power and 0.05 alpha [8]. 

Young Saudi females and males aged 20 to 30 years were recruited for participation in this study. Out of a total of 200 invited participants, 168 participants consented to participation, consisting of 84 Saudi male participants and 84 Saudi female participants, resulting in a participation rate of 84%.

The young Saudi participants with complete upper and lower dentition, with normal skeletal and dental relationship, normal positioning of the teeth without significant spacing, crowding, rotation, or tilting, and no missing teeth, caries, or prostheses in the anterior teeth were included in this study. Additionally, participants with acceptable healthy gingiva, no facial paralysis, and no abnormalities in the lips were included in this study.

Participants who were taking medication that caused gingival overgrowth, pregnant and lactating individuals, and those who were unwilling to participate in this study were excluded from the study.

Image capturing and video processing

In this study, a previous technique used for recording and processing dynamic smiles was employed [8,9].

Image Capturing

**Figure 1:**
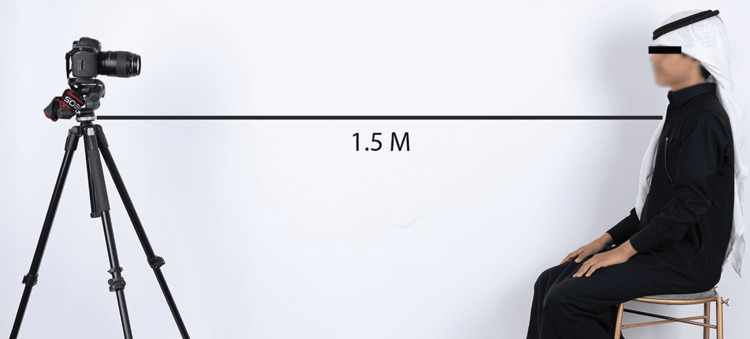
The camera is situated parallel to the head position, and the distance between the camera and the subject is 1.5 m.

Participants were positioned in an upright position. The camera (Canon EOS 600D, Tokyo, Japan) was set up parallel to the head and aligned with the Frankfort horizontal plane. The camera was situated 1.5 meters away from the participants and focused on the nose-chin region. A millimeter scale was placed near each subject’s right tragus for measurement calibration. A MacBook Pro (2.3 GHz, quad-core Intel Core i5 processor) placed directly behind the camera at eye level, played three short comedic films to induce natural dynamic smiles (Figure 1).

Image Processing

**Figure 2:**
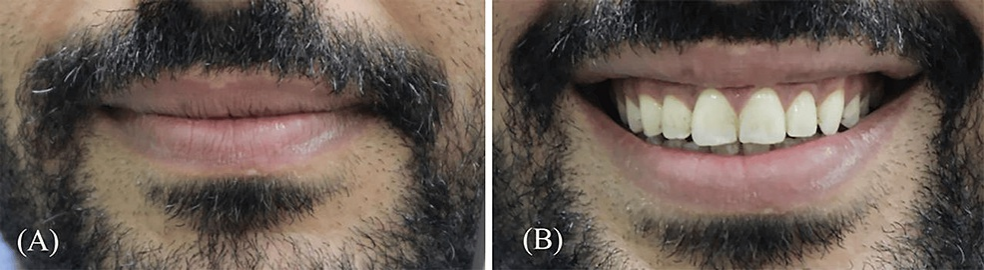
Images obtained from dynamic smile videos include: (A) a scale frame utilized to calculate the image enlargement ratio by comparing an actual 10 mm length to its image representation; (B) a full-smile frame that gathered data on smile characteristics, standardized for visual analog scale (VAS) evaluation.

Software for video editing (Sony Vegas Pro 20.0; Sony Creative Software Inc., Middleton, WI) was used for frame-by-frame analysis. Reference frames and full-smile frames were carefully selected for each participant, and the resulting images were transformed into JPEG files (Figure 2). These images were assigned code numbers and stored using a MacBook Pro (2.3 GHz, quad-core Intel Core i5 processor).

Image Editing and Standardization

**Figure 3:**
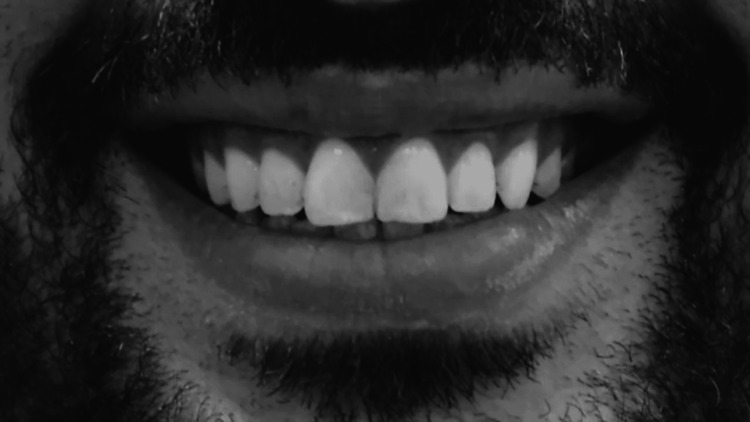
The final image format for visual analog scale (VAS) assessment.

Adobe Photoshop version 24 (Adobe Systems, San Jose, CA) was used to perform subsequent adjustments and edits to the full-smile photographs, involving cropping to remove potential distractions from surrounding facial elements. The standardized images were resized to dimensions of 5 × 3 inches, converted to grayscale, set to a saturation level of 2100, and set to a resolution of 70 dpi (Figure 3). These processed images were subsequently incorporated into Microsoft PowerPoint (Microsoft, Redmond, WA) to aid in the thorough evaluation of smile attractiveness.

Visual analog scale (VAS) attractiveness assessment of smiles

Smile images underwent visual assessments by 22 young Saudis (11 males and 11 females) aged 20-30 years. The purpose of the assessment was to choose between attractive and unattractive smiles. The raters' selection criteria included being Saudi nationals with no background in dental, esthetic, or artistic education and their willingness to participate in smile attractiveness assessments. After recruitment, the sex and age of the raters were obtained.

A questionnaire was used to explain the aim of this study, including the instructions for using the VAS, and to provide measurement pages for the VAS. The VAS utilized had a length of 100 mm, with raters marking between 0 (least attractive) and 100 (most attractive) to rate each image's attractiveness. Smile images, formatted in PowerPoint, were presented on a MacBook Air laptop (Apple, 1.1 GHz, dual-core Intel Core i3). Raters first reviewed all images before independently rating each smile's attractiveness by placing a mark on the VAS. Each image was shown for 10 seconds, during which raters could revise their scores. Then a manual caliber, in millimeters, was used to measure the marks from the left end of the VAS.

The average VAS scores for each of the 168 images were calculated from the assessments of 22 raters and then sorted from lowest to highest (*n* = 168). Subsequently, the images in the lowest and highest quartiles were classified as unattractive and attractive, respectively, each comprising 42 images (21 males and 21 females).

Smile characteristics

For the smile characteristics measurements, the Image Tool for Windows version 3.00 (University of Texas Health Science Center, San Antonio, TX) was used. The magnification ratio for each photograph was determined through a comparison of the actual length, as indicated on a calibrated scale, with the length measured in the image that included the scale reference.

**Figure 4:**
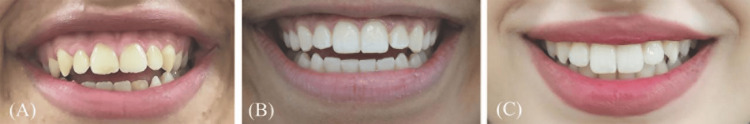
Anterior smile line categories. (A) High anterior smile line showing teeth and gingiva; (B) average anterior smile line showing teeth and interdental papillae; (C) low anterior smile line showing only teeth.

**Figure 5:**
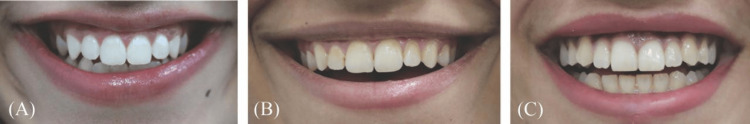
Smile arc categories: (A) parallel smile arc; (B) straight smile arc; and (C) reverse smile arc.

**Figure 6:**
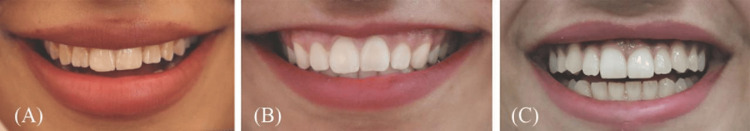
Upper lip curvature categories: (A) upward upper lip curvature, where the lip curves upwards; (B) straight upper lip curvature, where the lip maintains a straight line; and (C) downward upper lip curvature, where the lip curves downwards.

**Figure 7:**
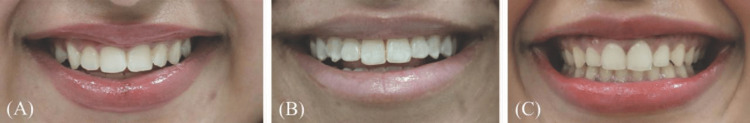
The most posterior tooth displayed while smiling: (A) more than 50% of the first premolar; (B) more than 50% of the second premolar; and (C) more than 50% of the first molar.

Six smile characteristics were rated. The anterior smile line is categorized as low, average, or high, based on tooth and gingiva visibility. An average smile line exhibits 75% to 100% visibility of maxillary teeth and gingival papillae, while a low smile line shows less than 75% visibility and a high smile line shows all maxillary teeth and gingiva (Figure 4) [20]. The smile arc is the parallelism between a line connecting the incisal edges of maxillary anterior teeth with the upper edge of the lower lip, which is categorized into three types. A parallel smile arc occurs when both lines are parallel. A straight smile arc is identified by a straight line connecting the incisal edges to the canine tips, without curvature. A reverse smile arc occurs when the maxillary incisal edges and canine points curve away from the lower lip's upper border (Figure 5) [21]. The upper lip curvature is classified as upward, straight, or downward. The upward upper lip curvature occurs when the mouth corner line is 1 mm or more above the lower border of the upper lip, the straight upper lip curvature occurs when the mouth corner is 1 mm or less below or above this line, and the downward lip curvature occurs when the lower border of the upper lip is more than 1 mm below the mouth corner (Figure 6) [8]. The most posterior teeth displayed are identified as teeth posterior to the maxillary canine that cover over 50% of the surface while smiling. These teeth are premolars or molars (Figure 7) [8]. The smile index is a measure of the ratio between the horizontal distance between the corners of the mouth (smile width) and the vertical distance from the bottom of the upper lip to the top of the lower lip (smile height) when a person smiles. It is computed by dividing the smile width by the smile height [8]. The smile symmetry examines the consistency of bilateral mouth corner movements both horizontally and vertically. It is determined by a specific formula that assesses the uniformity of these movements: (1-3) 1 (1-4)/(2-3) 1 (2-4) [8].

Assessment of intra-rater reliability

To assess intra-rater reliability, four randomly selected laypersons (two males and two females) were randomly chosen to redo their VAS evaluations after two weeks. Additionally, four trained and calibrated interns (two males and two females) evaluated the six smile characteristics. The reliability of these evaluations over a two-week interval was calculated using intra-class correlation coefficients (ICCs) for VAS data and continuous smile characteristics data (smile index and smile symmetry), as well as Cohen's kappa tests (κ) for the categorical data of smile characteristics.

Statistical analysis

The statistical analyses were performed using IBM SPSS Statistics for Windows (Version 26.0, IBM Corp., Armonk, NY). Due to the non-normal distribution of the data, the continuous variables VAS ratings, smile index, and smile symmetry values were presented as medians and interquartile ranges (IQRs), and categorical variables were presented as frequencies and percentages for each group based on the layperson’s ratings, attractive and unattractive groups.

Categorical variables included the anterior smile line, categorized as low, average, or high; the smile arc, categorized as parallel, straight, or reverse; upper lip curvature, classified as upward, straight, or downward; and the most posterior teeth displayed, identified as premolars or molars. To compare the categorical variables between attractive and unattractive groups, chi-square and Fisher's exact tests were employed.

The continuous variables included VAS ratings, smile index, and smile symmetry. The Mann-Whitney U test was used to compare the medians of these continuous variables between the attractive and unattractive groups. The study's significance level was set at *P* < 0.05.

Ethical considerations

The Ethics Committee at the University of Ha'il approved this study (Reference No. H-2023-056). This study was conducted in accordance Declaration of Helsinki. Participation in the research was explained to participants, and participation was voluntary, that is, the participants can withdraw at any time. All included participants gave consent through signatures. All participants whose pictures were used in the figures in this study were informed about and consented to the use of their images.

## Results

Intra-rater reliability findings

The intra-rater reliability analysis showed good consistency of VAS assessments among the four randomly selected laypersons, with ICC values of 0.661, 0.728, 0.859, and 0.94, respectively. Furthermore, the ICC for comparing the averages of the first and second assessments of the four raters was 0.737.

For the smile characteristics, the intra-rater reliability analysis demonstrated a high level of consistency between the two measures. Cohen's kappa tests yielded values ranging from 0.617 to 0.89, which indicate a significant level of agreement for the anterior smile line, smile arc, and upper lip curvature. In addition, the smile index had an ICC of 0.775, while the dynamic smile symmetry exhibited an ICC of 0.872, showing a high level of agreement.

VAS measurements

Table 1 shows VAS scores for all 168 participants, indicating gender-based differences in attractiveness assessment. Female participants had significantly higher attractiveness scores (*P* < 0.05), with a median of 55.5 (IQR = 40.8-66.0), compared to males with a median of 51.4 (IQR = 41.3-58.5).

**Table 1:** Median and interquartile ranges (IQR) of attractiveness visual analog scale (VAS) scores for all participants (n = 168). ^a^Mann-Whitney U test. ^*^Statistically significant at *P* < 0.05.

Gender	Median (IQR)	Minimum	Maximum	*P*-value ^a^
Males (*n *= 84)	51.4 (41.3-58.5)	15.4	80.5	0.035 *
Females (*n *= 84)	55.5 (40.8-66.0)	21.6	81.1

Table 2 shows the medians and IQR of VAS scores for the lowest 25th percentile unattractive and the highest 25th ratings of attractive smile groups (*n* = 84), stratified by gender. In the *unattractive* group, males' smiles were rated significantly more unattractive (median = 29.6, IQR = 25.0-34.2) than females (median = 35.0, IQR = 31.1-38.7) (*P* < 0.05). In the attractive group, females’ smiles were rated significantly more attractive (median = 71.0, IQR = 67.7-74.3) compared to males (median = 62.8, IQR = 61.0-71.6) (*P* < 0.05).

**Table 2:** Median and IQR of VAS scores for unattractive and attractive males and females (n = 84). ^a^Mann-Whitney U test. ^*^Statistically significant at *P* < 0.05. IQR, interquartile range; VAS, visual analog scale

Gender	Unattractive		Attractive	
	Median (IQR)	*P*-value^a^	Median (IQR)	*P*-value^a^
Males (*n *= 42)	29.6 (25.0-34.2)	0.006*	62.8 (61.0-71.6)	0.004*
Females (*n *= 42)	35.0 (31.1-38.7)		71.0 (67.7-74.3)	

Smile characteristics and attractiveness

Table 3 shows the distribution of smile characteristics between unattractive and attractive smiles (*n* = 84). The results show significant differences in the anterior smile line, with a higher prevalence of unattractive smiles compared to attractive ones (69.0% vs. 26.2%, *P* < 0.05). On the other hand, average or low smile lines occurred in 73.8% of the attractive smiles (*P* < 0.05). For smile arcs, parallel smile arcs were significantly perceived as attractive by 81%, compared to 31.3% of unattractive smiles, while non-parallel smile arcs were significantly predominant in unattractive smiles (*P* < 0.05). Smiles with a higher smile index, with a median of 5.1 (IQR = 4.7-6.1), were significantly more attractive than smiles with a lower median score of 4.5 (IQR = 3.8-5.0) (*P* < 0.05). No significant difference was found in upper lip curvature between the unattractive and attractive groups. The most posterior tooth smile characteristic did not show any significant difference between the groups. Furthermore, no significant difference was found in smile symmetry between unattractive and attractive smiles.

**Table 3:** Unattractive and attractive smile characteristics distribution of participants (n = 84). ^a^Chi-square test. ^b^Mann-Whitney U test. ^*^Statistically significant at *P* < 0.05. IQR, interquartile range

Smile characteristic	Classification	Unattractive, *n* (%)	Attractive, *n* (%)	*P*-value
Anterior smile line	High	29 (69.0%)	11 (26.2%)	0.00*^,^^a^
	Average/Low	13 (31.0%)	31 (73.8%)	
Lip curvature	Upward	16 (38.1%)	12 (28.6%)	0.35^a^
	Straight/Downward	26 (61.9%)	30 (71.4%)	
Smile arc	Parallel	14 (31.3%)	34 (81%)	0.00*^,^^a^
	Not parallel	28 (66.7%)	8 (19%)	
Most posterior tooth	Premolar	24 (42.9%)	18 (23.8%)	0.19^a^
	Molar	18 (57.1%)	24 (76.2%)	
Smile index	Median (IQR)	4.5 (3.8-5.0)	5.1 (4.7-6.1)	0.00*^,b^
Smile symmetry	Median (IQR)	1.0 (0.9-1.0)	1.0 (0.9-1.0)	0.95^b^

Table 4 shows the distribution of smile characteristics between unattractive and attractive smiles among males (*n* = 42) and females (*n* = 42). The results show significant differences in the anterior smile line, with a higher prevalence of high smile lines in unattractive smiles compared to attractive ones (66.7% vs. 14.3% in males, *P* < 0.05; 71.4% vs. 38.1% in females, *P* < 0.05), and the average or low smile lines were significantly perceived more attractive (85.7% in males, *P* < 0.05; 61.9% in females, *P* < 0.05). The smile arc also demonstrated a significant difference, with parallel arcs being significantly more common in attractive smiles (81% in both males and females; *P* < 0.05 for both males and females). Conversely, non-parallel smile arcs were significantly predominant in unattractive smiles. Smiles with a higher smile index were significantly more attractive across genders. For males, a more attractive smile had a median of 5.1 (IQR = 4.7-6.1), compared to a less attractive median of 4.2 (IQR = 3.8-4.9), and for females, an attractive smile had a median of 5.0 (IQR = 4.8-6.1) versus an unattractive median of 4.6 (IQR = 3.8-5.0), *P* < 0.05. However, for lip curvature, the most posterior tooth displayed, and smile symmetry, no significant differences were observed between the unattractive and attractive smile groups for both genders.

**Table 4:** Unattractive and attractive distribution of smile characteristics of males (n = 42) and females (n = 42). ^a^Fisher's exact test. ^b^Chi-square test. ^c^Mann-Whitney U test. ^*^Statistically significant at *P* < 0.05.

Smile characteristic	Classification	Males, *n* (%)		*P*-value	Females, *n* (%)		*P*-value
		Unattractive	Attractive		Unattractive	Attractive	
Anterior smile line	High	14 (66.7%)	3 (14.3%)	0.001*^,a^	15 (71.4%)	8 (38.1%)	0.03*^,b^
	Average/Low	7 (33.3%)	18 (85.7%)		6 (28.6%)	13 (61.9%)	
Lip curvature	Upward	6 (28.6%)	5 (23.8%)	0.72	10 (47.6%)	7 (33.3%)	0.34^b^
	Straight/Downward	15 (71.4%)	16 (76.2%)		11 (52.4%)	14 (66.7%)	
Smile arc	Parallel	5 (23.8%)	17 (81%)	0.001*^,a^	9 (42.9%)	17 (81%)	0.02*^,a^
	Not parallel	16 (76.2%)	4 (19%)		12 (57.1%)	4 (19%)	
Most posterior tooth	Premolar	15 (71.4%)	13 (61.9%)	0.51	9 (42.9%)	5 (23.8%)	0.19^b^
	Molar	6 (28.6%)	8 (38.1%)		12 (57.1%)	16 (76.2%)	
Smile index	Median (IQR)	4.2 (3.8-4.9)	5.1 (4.7-6.1)	0.001*^,c^	4.6 (3.8-5.0)	5.0 (4.8-6.1)	0.004*^,c^
Smile symmetry	Median (IQR)	1.0 (0.9-0.1)	1.0 (0.9-0.1)	0.64^c^	1.0 (0.9-1.0)	1.0 (0.9-1.0)	0.64^c^

## Discussion

This study provides evidence that young Saudi laypersons can reliably distinguish between attractive and unattractive smiles. This study included the dynamic smiles of 168 Saudi adults, consisting of 84 males and 84 females, which were assessed by 22 Saudi raters using a VAS. This study found that females' smiles were significantly rated as more attractive than males. Furthermore, the study identified specific smile characteristics that significantly impacted smile attractiveness, regardless of gender. These characteristics include anterior smile lines, smile arcs, and smile index. On the other hand, this study did not identify lip curvature, the most posterior displayed tooth during smiling, or whether smile symmetry had an impact on smile attractiveness for both genders.

Smile assessment should not be limited to static smile observations but should cover the dynamic range during its movement, as spontaneous smiles offer a more accurate evaluation of a patient's smile compared to posed or static expressions [9,22]. By capturing these natural expressions, dynamic assessment provides a nuanced understanding of aesthetic and functional requirements, which can significantly improve treatment outcomes and patient satisfaction. However, despite its obvious advantages, challenges remain in terms of practicality, resource allocation, and lack of standardized methods [22]. To address these challenges and accurately assess smile characteristics, incorporating 3D modeling and videography emerges as a promising approach. This technology enables the quantification of precise thresholds for smile features, thereby providing a solid foundation for aesthetic diagnosis and tailor-made treatment planning. The use of dynamic analysis, supported by advanced imaging techniques, offers a comprehensive strategy to overcome practical limitations and improve the precision of smile assessments [5,9,23].

The VAS is a widely employed tool for assessing subjective aesthetic perception. It is commonly used in epidemiological and clinical studies [24]. In dentistry, VAS is the instrument most commonly used to assess smile aesthetic perception [25]. This scale is reliable for assessing smile and dental attractiveness, validating its effectiveness in subjective evaluations [26]. In addition, its quantitative nature ensures the reliability and validity of subjective assessments, making it economically efficient and adaptable to various environments [27]. However, VAS is not without limitations. It may not be intuitively understandable for all respondents, particularly across different age groups or those with physical or visual impairments [28].

In this study, smiles characterized by average or low anterior smile lines were rated as more attractive across genders. This is consistent with findings from Saudi studies, where smiles showing more maxillary gingiva were rated as the least attractive [16,17,19,29]. Such findings also align with earlier studies indicating that excessive gingival display compromises esthetics [8,12,30-35]. However, one study presented a contrasting viewpoint, suggesting a preference for higher gingival lines [13]. These findings highlight the importance of considering the anterior smile line during diagnosis and treatment planning. Clinicians should address etiologies such as altered passive eruption and short upper lips through periodontal plastic surgery, orthodontics, or lip repositioning or augmentation to optimize gingival height display based on patient preferences and cultural background [36]. However, quantitative measures are necessary to establish precise thresholds at which gingival display transitions from enhancing to detracting smile attractiveness across diverse demographic groups.

In contrast with previous studies, the upper lip curvature did not significantly impact attractiveness ratings in this Saudi sample for both genders. Most of these studies showed preferences for upward, straight, and to a lesser extent reverse smile curvature, suggesting that upper lip curvature can be perceived as attractive in various forms [8,34,37-40]. This finding highlights that attractiveness might vary widely across different cultures and individuals. Considering the inability to modify upper lip curvature through dental procedures, clinicians must set realistic expectations with their patients [8,41].

Our study findings reveal that the parallel smile arc was perceived as more attractive across all genders, supporting the belief that a parallel smile arc enhances smile attractiveness. This finding is in line with previous research [5,34,42-44]. Interestingly, the Saudi female college students were more critical of consonant smile arcs, a perspective that coincides with our observations [18]. Contrarily, several studies argue that the parallel smile arc does not significantly affect smile attractiveness [8]. This discrepancy underscores the complexity of smile perception and highlights the importance of considering the parallelism of the smile arc about the lower lip when planning restorative and orthodontic treatments [5]. Utilizing mock-ups or virtually altered smiles could be instrumental in addressing these considerations, thereby facilitating more tailored and effective treatment outcomes.

This study found no significant impact on the attractiveness of posterior teeth during dynamic smiling in both genders, a finding that aligns with similar previous studies [8,34]. It also found that participants with a smile index of 5 or above were perceived as significantly more attractive, which is consistent with previous studies that identified a preference for a smile index exceeding 5 [8,41]. Additionally, a previous study suggested that enhanced smile indices might be attributed to the practice of smile exercises [41]. For the impact of smile symmetry on attractiveness, this study found no significant differences. However, smiles nearing a symmetry value of 1 were preferred, confirming the findings of previous similar studies that highlighted the attractiveness of near-perfect smile symmetry [8,34].

This study indicates that laypersons' preferences for smile characteristics did not differ by gender, as supported by previous research [5,8]. Despite the finding that there were no gender-based differences in smile attractiveness, it acknowledges that cultural or ethnic factors might affect gender perceptions of aesthetic smiles [45].

A standard technique of dynamic smile assessments was utilized in this study to rate attractiveness, which can provide accurate and natural smile assessments. It also might fill the knowledge gaps providing a valued understanding for dental professionals to adapt aesthetic treatment plans for their patients in this cultural setting. However, this study had limitations; the non-probability sampling technique could compromise the generalization of its findings. In addition, the subjectivity of the VAS measurement tool might cause a potential bias. Furthermore, the age group of the participants could prevent the application of the findings of this study to other age groups.

This study provides foundational data on Saudi smile preferences by evaluating several variables that may influence the perception of dynamic smile attractiveness by laypersons for the first time. However, comprehensive research with expanded populations is essential to explain the complex relationships between the various determinants of smile attractiveness. Future studies should include a broader and more diverse group of laypersons from various socio-cultural backgrounds to verify the persistence of these trends.

## Conclusions

Within the limitations of the present study, it has been successfully demonstrated that young Saudi laypersons possess a reliable ability to differentiate between attractive and unattractive smiles. The analysis of VAS confirmed gender-based differences in perceived smile attractiveness, with female smiles generally rated higher than male smiles. However, for the impact of smile characteristics on perceived attractiveness, no significant gender differences were found. The smile characteristics, notably average or low anterior smile lines, parallel smile arcs, and higher Smile Index values, were identified as significantly impacting the attractiveness of smiles in both genders.

This study suggested that cultural or ethnic standards can play a critical role in smile perceptions indicating the potential for further research to explore demographic impacts on smile aesthetics in different cultures around the world that can provide scientific evidence that enables dental professionals to customize treatment plans to individual smile patterns and lip shapes.
